# Multidimensional analysis of immune cells from COVID-19 patients identified cell subsets associated with the severity at hospital admission

**DOI:** 10.1371/journal.ppat.1011432

**Published:** 2023-06-13

**Authors:** Sergio Gil-Manso, Diego Herrero-Quevedo, Diego Carbonell, Marta Martínez-Bonet, Esther Bernaldo-de-Quirós, Rebeca Kennedy-Batalla, Jorge Gallego-Valle, Rocío López-Esteban, Elena Blázquez-López, Iria Miguens-Blanco, Rafael Correa-Rocha, Vanessa Gomez-Verdejo, Marjorie Pion

**Affiliations:** 1 Advanced ImmunoRegulation Group, Gregorio Marañón Health Research Institute (IiSGM), General University Hospital Gregorio Marañón, Madrid, Spain; 2 Signal Processing and Communications Department, University Carlos III de Madrid, Leganés, Madrid, Spain; 3 Department of Hematology, General University Hospital Gregorio Marañón (HGUGM), Madrid, Spain; 4 Gregorio Marañón Health Research Institute (IiSGM), Madrid, Spain; 5 Laboratory of Immune-Regulation, Gregorio Marañón Health Research Institute (IiSGM), General University Hospital Gregorio Marañón, Madrid, Spain; 6 Gastroenterology—Digestive Service, General University Hospital Gregorio Marañón, Network of Hepatic and Digestive Diseases (CIBEREHD), Carlos III Health Institute (ISCIII), Madrid, Spain; 7 Emergency Department, General University Hospital Gregorio Marañón, Madrid, Spain; Duke-National University of Singapore, SINGAPORE

## Abstract

**Background:**

SARS-CoV-2 emerged as a new coronavirus causing COVID-19, and it has been responsible for more than 760 million cases and 6.8 million deaths worldwide until March 2023. Although infected individuals could be asymptomatic, other patients presented heterogeneity and a wide range of symptoms. Therefore, identifying those infected individuals and being able to classify them according to their expected severity could help target health efforts more effectively.

**Methodology/Principal findings:**

Therefore, we wanted to develop a machine learning model to predict those who will develop severe disease at the moment of hospital admission. We recruited 75 individuals and analysed innate and adaptive immune system subsets by flow cytometry. Also, we collected clinical and biochemical information. The objective of the study was to leverage machine learning techniques to identify clinical features associated with disease severity progression. Additionally, the study sought to elucidate the specific cellular subsets involved in the disease following the onset of symptoms. Among the several machine learning models tested, we found that the Elastic Net model was the better to predict the severity score according to a modified WHO classification. This model was able to predict the severity score of 72 out of 75 individuals. Besides, all the machine learning models revealed that CD38+ Treg and CD16+ CD56neg HLA-DR+ NK cells were highly correlated with the severity.

**Conclusions/Significance:**

The Elastic Net model could stratify the uninfected individuals and the COVID-19 patients from asymptomatic to severe COVID-19 patients. On the other hand, these cellular subsets presented here could help to understand better the induction and progression of the symptoms in COVID-19 individuals.

## Author summary

The continuous threat of SARS-CoV-2 or other potential future viral pandemics have made necessary predictive methods for patient management, especially for patients that will develop severe clinical conditions once hospitalized. Since the beginning of the COVID-19 pandemic, large amounts of clinical and immunological data have been obtained. In this manuscript, we utilized several machine learning models based on clinical data and immune cellular subsets obtained by flow cytometry to predict those individuals that could progress to severe COVID-19. Non-infected individuals were compared to those presenting symptoms, tested at the Emergency Service, and followed until their hospital discharge or death due to the SARS-CoV-2 infection. Here, the Elastic Net model permitted us to determine variables that could predict individuals that will present more severe disease progression. This work could serve two purposes. Firstly, it could help clinicians to stratify individuals who are at risk of developing severe COVID-19. Secondly, it could assist researchers in determining the specific immune subsets implicated in the severity of the disease.

## Introduction

The SARS-CoV-2 was a novel coronavirus that emerged in December 2019, and since the beginning of 2020 was responsible for the pandemic of COVID-19 (Coronavirus Disease 2019). According to the World Health Organization, in March 2023, more than 760 million infections and 6.8 million deaths were reported associated with SARS-CoV-2 infection worldwide (data obtained from World Health Organization: https://covid19.who.int/).

During the first COVID-19 wave, according to the Chinese Center for Disease Control and Prevention report, 81% of individuals presented a mild disease, 14% severe disease, 5% critical and a case-fatality rate of 2.3% [1]. These findings were comparable to what was observed in another study in the United States of America, with 14% of individuals hospitalized, 2% of admission to Intensive Care Unit (ICU) and 5% deaths of total infected individuals [2]. However, the clinical spectrum of infected patients has changed over the pandemic (after vaccination, reinfections, and regarding the SARS-CoV-2 variants). According to a study about the Omicron variant, 46.7% of infected individuals were asymptomatic [3]. Besides, other studies have observed that the individuals infected with Omicron had a lower risk of severe COVID-19, ICU admission and mortality in comparison with the Delta variant [4–11], Alpha variant [4, 5] and Wuhan original strain [12]. However, symptomatic infections and deaths related to COVID-19 are still being reported and are still a problem for society and healthcare systems, especially in managing hospitalized patients.

A meta-analysis work reviewed 152 studies and identified six symptoms with the highest prevalence related to COVID-19: fever (in 58.66% of infected individuals), cough (54.22%), dyspnea (30.82%), malaise (29.75%), fatigue (28.16%) and sputum (25.33%) [13]. However, other symptoms are less prevalent but can be implicated in the severity of the COVID-19. Therefore, predicting those individuals that could need intensive care is still necessary because the spread of the virus is still responsible for thousands of daily infections. This spreading is partially responsible for the saturation of the healthcare systems and for preventing the proper treatment of patients once hospitalized, which could lead to the deterioration of the health and, eventually, fatality [14]. Moreover, the availability of tools to decipher the immune features that contribute to severe disease progression is pivotal in understanding the disease’s mechanisms and its immune response. Thus, tools that can predict severity in early infected individuals at the moment they go to the Hospital Emergency Department and explain why some patients are expected to have severe COVID-19 could be useful. This could be important not only for SARS-CoV-2 but also for future viral-associated respiratory pathologies, and to devote greater healthcare efforts to improve their clinical situation.

Several groups developed machine learning models (LASSO [15, 16], XGBoost [17, 18] or Random Forest [17, 18]) to predict severity in infected patients and already determined key features correlated with COVID-19 severity, such as, lymphopenia [16, 19–21], and high levels of ferritin [19], C-Reactive Protein [18–20], lactate dehydrogenase [14–16, 19, 20], neutrophils [16, 21, 22], D-Dimer [16, 18, 22, 23], IL-6 [17, 24], IL-10 [17, 24], and some features of quantitative computed tomography (CT) [16, 25]. However, although the SARS-CoV-2 infection directly influences the immune system, no study has used immune subsets to develop a machine learning model to predict severity, except for neutrophils and lymphocyte counts.

Here we propose the Elastic Net model as a new machine-learning solution to stratify patients according to their expected severity. Additionally, this model has the potential to aid researchers in elucidating the significance of specific immune subsets that have been linked to the severity of the disease. As a result, this approach highlights 2 specific immune subsets highly implicated in the prediction of severity which could help expand the range of known predictor features.

## Results

### Selection of the better machine learning model and clinical characteristics that are associated with the more robust model

The data utilized in the models was collected within the first 18 hours since the admission to the Emergency Service. The variables collected, which are listed in S1 Table, included personal characteristics, symptoms experienced since the onset of infection, biochemical parameters, cytokine levels measured in plasma, and the percentage and absolute numbers of immune subsets measured in peripheral blood via flow cytometry (S2 Table). After processing the data as described in the Materials and Methods section, we tested several models, including Elastic-net, linear GP with ARD, Lasso, Random Forest and Linear Ridge Regression, to determine which one best predicted the COVID severity across different patients and detected features related to different cohorts. We calculated the average for the mean absolute error (MAE), mean squared error (MSE) and R2 score for all predictions produced by each model. The results showed an improved LOO performance in terms of R2 for the Elastic Net model, with an MAE of 0.657, an MSE of 0.870, and an R2 score of 0.723 (Table 1).

**Table 1:** Characteristics of the machine learning models’ results. The table shows the Mean Absolute Error (MAE), Mean Squared Error (MSE) and R2 score for those machine learning models that yielded an R2 score over 0.6.

	MAE	MSE	R2
GP_linear_ARD	0.748	1.165	0.630
Elastic_Net	0.657	0.870	0.723
Lasso	0.770	1.020	0.676
Random_Forest_Regressor	0.862	1.259	0.600
Kernel_Ridge_Linear	0.797	1.201	0.618

**Fig 1:**
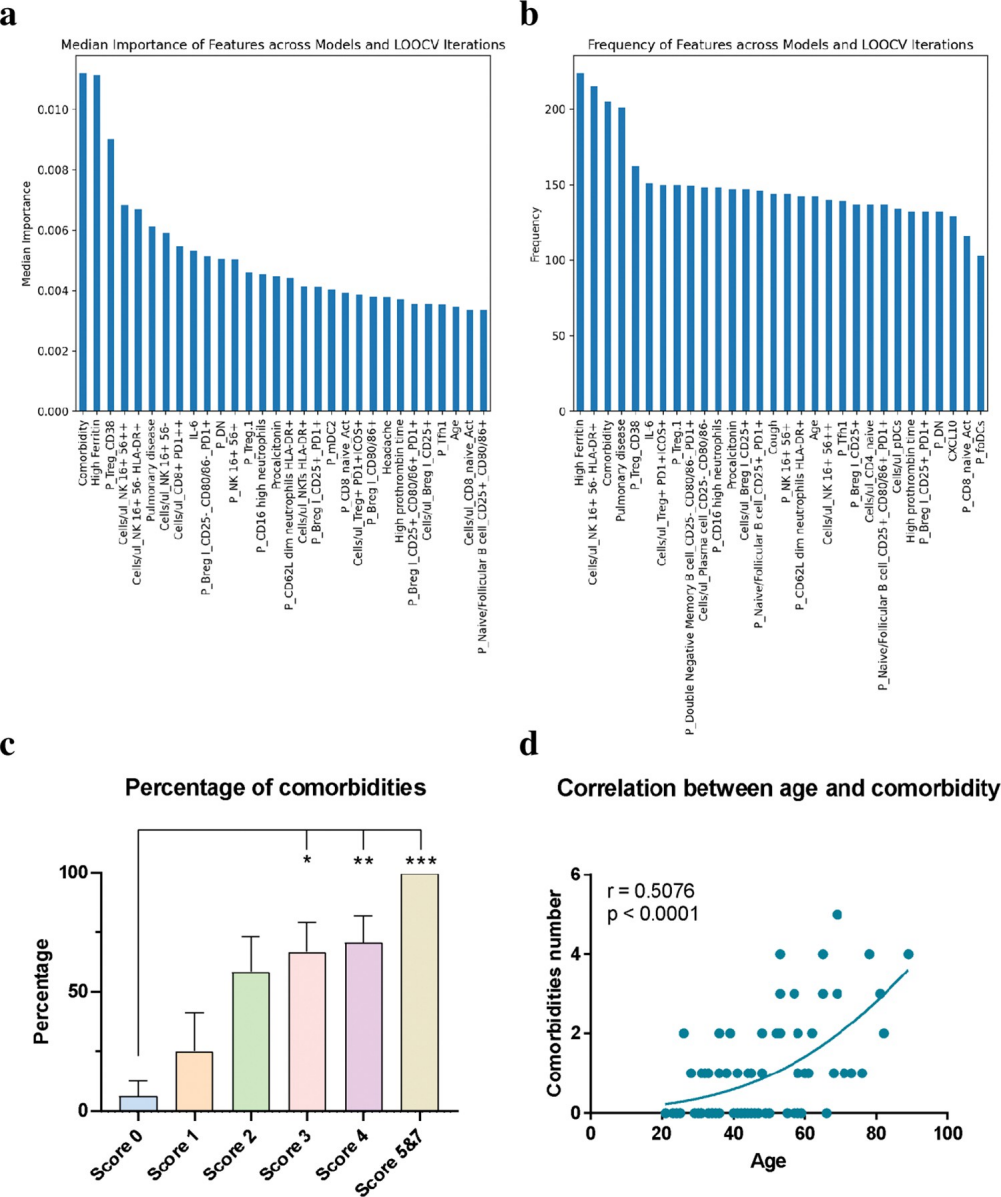
Most important features among the machine learning models and relevance of comorbidities. Median importance (a) and frequency of apparition of features (b) across all the machine learning models tested. The presence of comorbidities is one of the most important features, and the percentage of comorbidities in each Severity Score group indicates a positive correlation (c). Correlation between age and the frequency of comorbidities (d). Kruskal-Wallis test and Dunn’s correction were done in graph (c). Shapiro-Wilk test was done for the normality test, and Spearman correlation was done in graph (d). *p < 0.05, **p < 0.01, *p < 0.001.

Moreover, analyzing these 5 models that yielded an R2 score over 0.6, we identified the features that consistently were considered most relevant (with the greatest weight) across all these models, detailed in Fig 1A and 1B. Fig 1A refers to which features have a greater impact on the model’s accuracy, and it is calculated from multiple models and cross-validation iterations to obtain a more precise and reliable estimate. Fig 1B presents a measure of which features are more consistent and appear more frequently in the different models and the LOO iterations. This information can be useful for identifying important and reliable features in the dataset and for improving the accuracy of the model by removing features that are not relevant or consistent. Besides, these results confirm that linear models are a good choice for this problem providing similar or better results than nonlinear ones. In summary, comorbidities and high ferritin levels, which were already described as related to COVID-19 severity [19, 26, 27], were associated in all the machine learning models tested as highly relevant for the severity prediction. On the other hand, the frequency of CD38+ Treg and the absolute number of CD16+ CD56neg HLA-DR+ NK were also determined to predict severity adequately. Both subsets were also identified as relevant when a sensitivity analysis was conducted (S1 Fig). Out of the 575 features studied, both subsets were among the 30 most relevant features.

### Patient’s characteristics and importance of comorbidities in the COVID-19 severity

Among all the variables studied, we focused on the age of individuals since it is well-studied that older people presented a higher severity of COVID-19 [1, 15, 21, 28, 29]. As expected, our cohort analysis revealed that the machine learning model and sensitivity analysis identified age as a relevant factor for predicting disease severity (Fig 1A and 1B and S1 Fig). The median age was strongly correlated with the Severity Score, with a higher severity in older people. Pearson correlation between age and Severity Score indicated an r coefficient of 0.4617 (*p*-value < 0.0001, Table 2).

**Table 2:** Detailed characteristics of individuals regarding their Severity Scores. For each Severity Score group, it is detailed the number of individuals, age, sex, and a list of comorbidities related to SARS-CoV-2 infection. Symptoms indicate the number of symptoms developed by each individual since the beginning of the infection to the admission into the Emergency Service. Time in days between symptoms onset and hospitalisation and days between hospitalisation and hospital discharge. Chi-squared test was used for the analysis of gender. For age, Pearson correlation and p-value, and for comorbidities, days between symptoms onset and hospitalisation, and days between hospitalisation and hospital discharge/death, Spearman correlations and p-values are detailed.

	Score 0	Score 1	Score 2	Score 3	Score 4	Score 5&7	P value
**Number of individuals**	16	8	12	15	17	7	
**Age (years)**	41.81 (± 3.25)	27.87 (± 2.37)	43.25 (± 3.73)	53.46 (± 4.52)	51.70 (± 3.62)	64.71 (± 4.51)	r = 0.4617 *p*-value < 0.0001
Sex **(male/female)**	5/11	3/5	3/9	8/7	11/6	3/4	0.202
**Ethnic**							
European	16	5	3	8	6	5	
Latin	0	3	8	6	10	2	
African	0	0	1	1	1	0	
**Comorbidities, (%)**	1 (6.25)	2 (25)	7 (58.33)	10 (66.67)	12 (70.58)	7 (100)	r = 0.5585 *p*-value < 0.0001
Obesity	1	1	2	4	4	3	
Dyslipidemia	0	0	3	4	6	2	
Arterial Hypertension	0	0	2	5	5	1	
Diabetes	0	0	1	0	2	1	
Pulmonary disease	1	1	1	3	4	2	
Hepatic disease	0	0	3	1	0	1	
Heart disease	0	1	0	1	4	1	
Cancer	0	0	0	1	1	2	
**Symptoms**							
Low fever (<38°C)	-	5	11	9	12	5	
Fever (≥38°C)	-	5	9	6	9	1	
Cough	-	2	9	10	10	3	
Throat pain	-	3	1	1	3	1	
Dyspnoea	-	2	4	8	9	5	
Asthenia	-	2	4	5	6	2	
Headache	-	5	5	4	7	2	
Diarrhoea	-	3	5	4	5	3	
Nausea	-	1	2	2	2	1	
Vomiting	-	1	2	4	4	1	
Myalgia	-	2	8	3	8	1	
Ageusia	-	1	3	3	1	0	
Anosmia	-	1	2	2	3	0	
Time (**Days +/- SEM) between symptoms onset and hospitalisation**	-	-	8.91 +/- 2.49	6.47 +/- 1.25	5.81 +/- 0.80	5.14 +/- 1.01	r = -0.1562 *p*-value = 0.2838
Time (**Days +/- SEM) between hospitalisation and hospital discharge/death**	-	-	5.17 +/- 1.19	7.67 +/- 1.27	11.24 +/- 1.54	30.57 +/- 11.38	r = 0.5400 *p*-value < 0.0001

It is also described that comorbidities such as obesity, dyslipidemia, or previous pulmonary diseases are factor risks in the severity of COVID-19 [26, 27]. We then studied the percentage of volunteers presenting at least one comorbidity considered as a factor risk previous to the infection by the SARS-CoV-2. We observed that despite 25% of volunteers in Score 1 presented at least one comorbidity, more than half showed comorbidities in the rest of the score groups. The frequency of individuals presenting comorbidities reached 100% of volunteers in Score 5&7 (Score 2; 58.33%, Score 3; 66.67%, Score 4; 70.58%, Fig 1C and Table 2). Also, the Spearman correlation between the percentage of volunteers with comorbidities and the Severity Score was r = 0.5585 (p < 0.0001, Table 2). Besides, a highly positive correlation between age and the number of comorbidities was observed as expected (r = 0.5076, p < 0.0001, Fig 1D). However, here we couldn’t conclude if comorbidities, age or a combination of both are directly implicated in the severity of COVID-19. Indeed, older individuals presented more comorbidities. However, despite the strong correlation between both features, the presence of comorbidities has the highest weight in all the machine learning models tested than age (Fig 1A and 1B).

### The machine learning model allowed us to predict SARS-CoV-2 infected individuals regarding their Severity Score

Among all machine learning models tested, the Elastic Net model was identified as the best machine learning model to predict the Severity Scores of the different patients. Compared to other tested models, the Elastic Net model was the one that best adapted to our data, presenting an R2 score of 0.723 and an MAE of 0.675 (Table 1).

**Fig 2:**
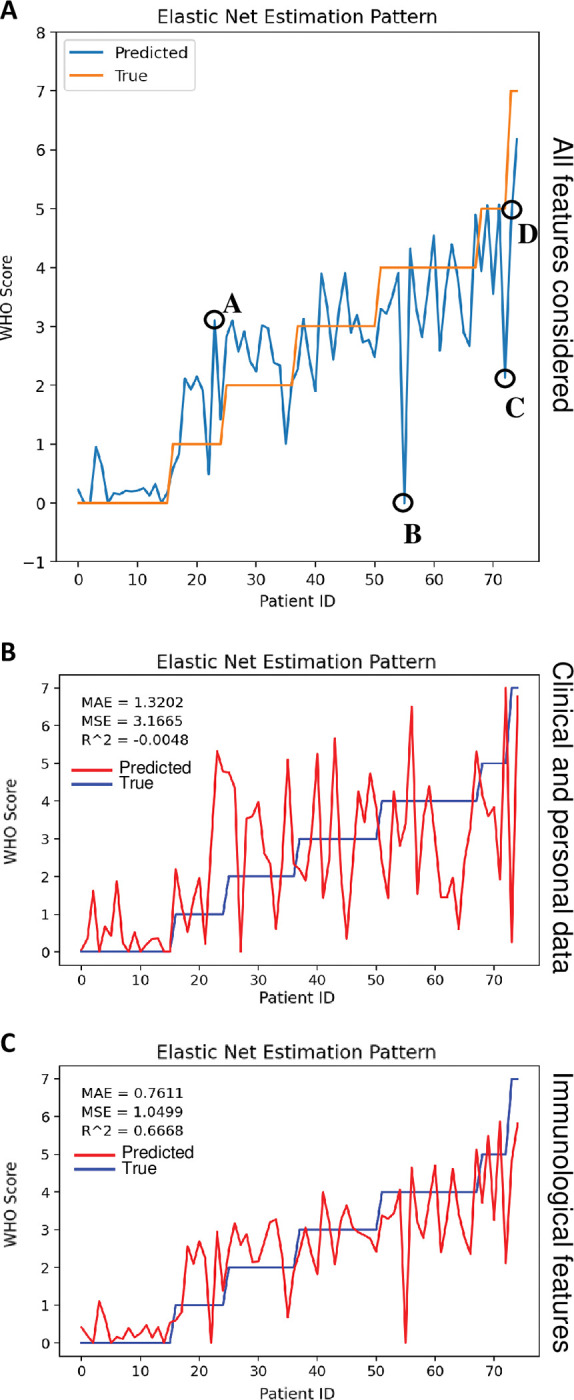
Elastic net model to predict Severity Score. (a) Severity Score predictions by Elastic Net model (light blue) and the ground truth values (orange) on unbalanced and rounded data at each interaction. Misclassified individuals are indicated with letters from A to D. (b) Severity Score predictions by Elastic Net model (red) and the ground truth values (dark blue) on unbalanced and rounded data at each interaction including only the clinical and personal data. (c) Severity Score predictions by Elastic Net model (red) and the ground truth values (dark blue) on unbalanced and rounded data at each interaction including only the immunological data. MAE for the mean absolute error, MSE for the mean squared error, and R^2 score were calculated for all predictions.

Applying the Elastic Net model to the whole data of all the patients, we could observe that the model correctly predicted the COVID-19 Severity Score for most of the individuals. In Fig 2A, the orange line indicates the actual value attending to the patient’s oxygen therapy requirement, while the blue line indicates the predicted value provided by the Elastic Net model. Although most of the patients were accurately predicted, we considered that those with a variation of ± 1.5 in the score were misclassified, resulting in 4 individuals out of 75 individuals. Patient A was initially classified in Score 1, but the model calculated a Score of 3.1. However, this patient was misclassified because he received an oxygen therapy of 3L, so his real score should be 3, according to what was obtained by the model. The other three patients misclassified by the model were: Patient B had a real Score of 4 but the model classified him as the Score of 0, Patient C had a Score of 5, but the model provided a Score of 2.13, and Patient D was classified with a Score of 4.74, when his real Score was 7. Therefore, out of the 75 individuals, only 3 individuals were predicted in another Score group, resulting in an error of the machine learning model of 4%. This incorrect classification could be explained by the heterogeneity of the COVID-19-related symptoms and immune subsets.

Therefore, the Elastic Net model would allow for predicting the severity of the COVID-19 infection from the very moment they were admitted to the emergency department. Nevertheless, because immune subset analysis is not a routine analytic in hospitals, we were interested in running the Elastic Net model using only clinical features and personal data (Fig 2B), as well as only immunological features (Fig 2C). It was striking to observe that clinical features and personal data alone were not able to predict severity, as the model using only these variables had an R^2 of around zero. Conversely, the immunological features provided good prediction (R^2 = 0.66), but it was the combination of immunological and clinical variables that yielded the best performance (R^2 = 0.72). Therefore, this predictive model may be more appropriate for research on the importance of initial immune cell subsets and cytokine concentrations at the onset of the disease, in conjunction with clinical/personal data, rather than for detecting patients from a clinical perspective.

When verifying how cytokines and biochemical markers were modulated regarding the severity score, we could observe that PCR, procalcitonin, IL-6, IL-8, TNF-α, IL-10, IL-13 and CXCL10 were positively correlated with the severity score, as expected (Table 3).

**Table 3:** Levels of classical COVID-19 biomarkers with the highest importance among the machine learning models. Mean ± SEM levels are reported for biochemical markers including C-reactive protein, D-dimer, ferritin, procalcitonin, lactate dehydrogenase (LDH), fibrinogen, prothrombin time, alanine aminotransferase (ALT), and aspartate aminotransferase (AST), as well as cytokines such as IL-1β, IL-6, IL-8, TNF-α, CCL2, IL-12p70, IL-10, IL-13, IL-17A, CXCL10, GM-CSF and IFN-γ for each Severity Score group. Some information is missing for biochemical markers in Score 0 and Score 1 because they were not measured during their admission to the Emergency Service. Pearson correlations were performed between biomarkers and severity score. p = p-value and r = correlation coefficient. In bold, when p < 0.05 which is considered as significant.

	Score 0	Score 1	Score 2	Score 3	Score 4	Score 5&7	Spearman r
**Number of individuals**	16	8	12	15	17	7	
C-reactive protein (mg/dL) [0–0.5]	-	0.4 (± 0.14)	8.84 (± 2.24)	6.05 (± 1.17)	7.52 (± 1.46)	14.83 (± 2.91)	r = 0.3459 **p = 0.0097**
D-dimer (ng/mL) [0–250]	-	171.50 (± 59.80)	290.45 (± 58.09)	266.07 (± 45.65)	447.94 (± 200.64)	287.33 (± 81.87)	r = -0.0447 p = 0.7600
Ferritin (μg/L) Range [19–285]	-	-	559.57 (± 186.70)	453.08 (± 123.97)	642.44 (± 202.75)	1245.83 (± 640.51)	r = 0.1648 p = 0.3032
Procalcitonin (μg/ml) Range [< 0.5]	-	-	0.09 (± 0.03)	0.05 (± 0.01)	0.07 (± 0.01)	0.97 (± 0.80)	r = 0.3399 **p = 0.0147**
Lactate dehydrogenase (LDH, U/L) Range [135–219.5]	-	182.50 (± 61.06)	288.44 (± 47.13)	291.15 (± 32.82)	275.19 (± 23.81)	331.67 (± 54.16)	r = 0.1851 p = 0.2180
Fibrinogen (mg/dL) Range [150–450]	-	438.75 (± 119.17)	733.42 (± 39.62)	675.53 (± 25.93)	685.00 (± 52.40)	783.57 (± 69.53)	r = 0.2084 p = 0.1304
Prothrombin time (s) [10.5–13.5]	-	11.67 (± 3.50)	13.18 (± 0.30)	13.31 (± 0.39)	12.96 (± 0.23)	23.20 (± 9.85)	r = 0.1241 p = 0.3712
Alanine aminotransferase (ALT, U/L) [5–36]	-	23.00 (± 6.96)	42.33 (± 9.51)	57.13 (± 10.51)	46.59 (± 10.37)	43.57 (± 10.00)	r = 0.08535 p = 0.5395
Aspartate aminotransferase (AST, U/L) [10–34]	-	-	112.67 (± 36.65)	63.60 (± 12.91)	60.13 (± 13.58)	69.33 (± 22.38)	r = 0.0858 p = 0.6900
IL-1β (pg/ml)	0.54 (± 0.05)	0.73 (± 0.09)	0.65 (± 0.15)	0.51 (± 0.04)	0.61 (± 0.09)	0.56 (± 0.08)	r = 0.01743 p = 0.8820
IL-6 (pg/ml)	1.20 (± 0.19)	5.85 (± 1.25)	26.45 (± 11.44)	16.94 (± 3.69)	25.08 (± 9.78)	75.80 (± 49.94)	r = 0.5942 **p < 0.0001**
IL-8 (pg/ml)	6.67 (± 0.60)	57.43 (± 25.93)	51.58 (± 43.42)	10.79 (± 1.03)	13.31 (± 2.46)	17.20 (± 2.37)	r = 0.3235 **p = 0.0046**
TNF-α (pg/ml)	7.71 (± 0.40)	14.97 (± 1.58)	11.92 (± 1.68)	13.56 (± 1.22)	13.15 (± 1.78)	13.00 (± 0.91)	r = 0.3547 **p = 0.0018**
CCL2 (pg/ml)	188.21 (± 17.21)	943.81 (± 287.20)	321.93 (± 87.87)	361.47 (± 67.87)	428.84 (± 136.19)	368.23 (± 93.39)	r = 0.1537 p = 0.1880
IL-12p70 (pg/ml)	1.29 (± 0.02)	1.74 (± 0.35)	1.67 (± 0.22)	1.49 (± 0.11)	1.36 (± 0.06)	1.60 (± 0.26)	r = 0.0906 p = 0.4391
IL-10 (pg/ml)	2.38 (± 0.11)	4.98 (± 1.14)	7.98 (± 1.16)	11.72 (± 2.11)	18.43 (± 7.86)	17.01 (± 3.16)	r = 0.7046 **p < 0.0001**
IL-13 (pg/ml)	43.41 (± 18.04)	21.40 (± 11.13)	14.78 (± 4.98)	10.09 (± 1.35)	12.48 (± 3.02)	9.90 (± 2.63)	r = -0.2651 **p = 0.0225**
IL-17A (pg/ml)	2.10 (± 0.00)	2.50 (± 0.41)	2.62 (± 0.52)	2.32 (± 0.13)	2.29 (± 0.10)	2.39 (± 0.19)	r = 0.2043 p = 0.0808
CXCL10 (pg/ml)	89.28 (± 10.13)	1120.57 (± 281.15)	943.53 (± 163.75)	1037.47 (± 154.00)	1149.82 (± 142.42)	1629.43 (± 137.73)	r = 0.6376 **p < 0.0001**
GM-CSF (pg/ml)	2.43 (± 0.29)	1.71 (± 0.15)	11.03 (± 4.99)	9.41 (± 4.48)	5.09 (± 0.90)	2.00 (± 0.27)	r = 0.2569 **p = 0.0261**
IFN-γ (pg/ml)	0.68 (± 0.07)	3.13 (± 1.02)	25.89 (± 8.78)	13.41 (± 3.39)	7.26 (± 1.90)	7.45 (± 2.30)	r = 0.4077 **p = 0.0003**

### An unsupervised analysis identified a metacluster related to CD38+ Treg cells

**Fig 3:**
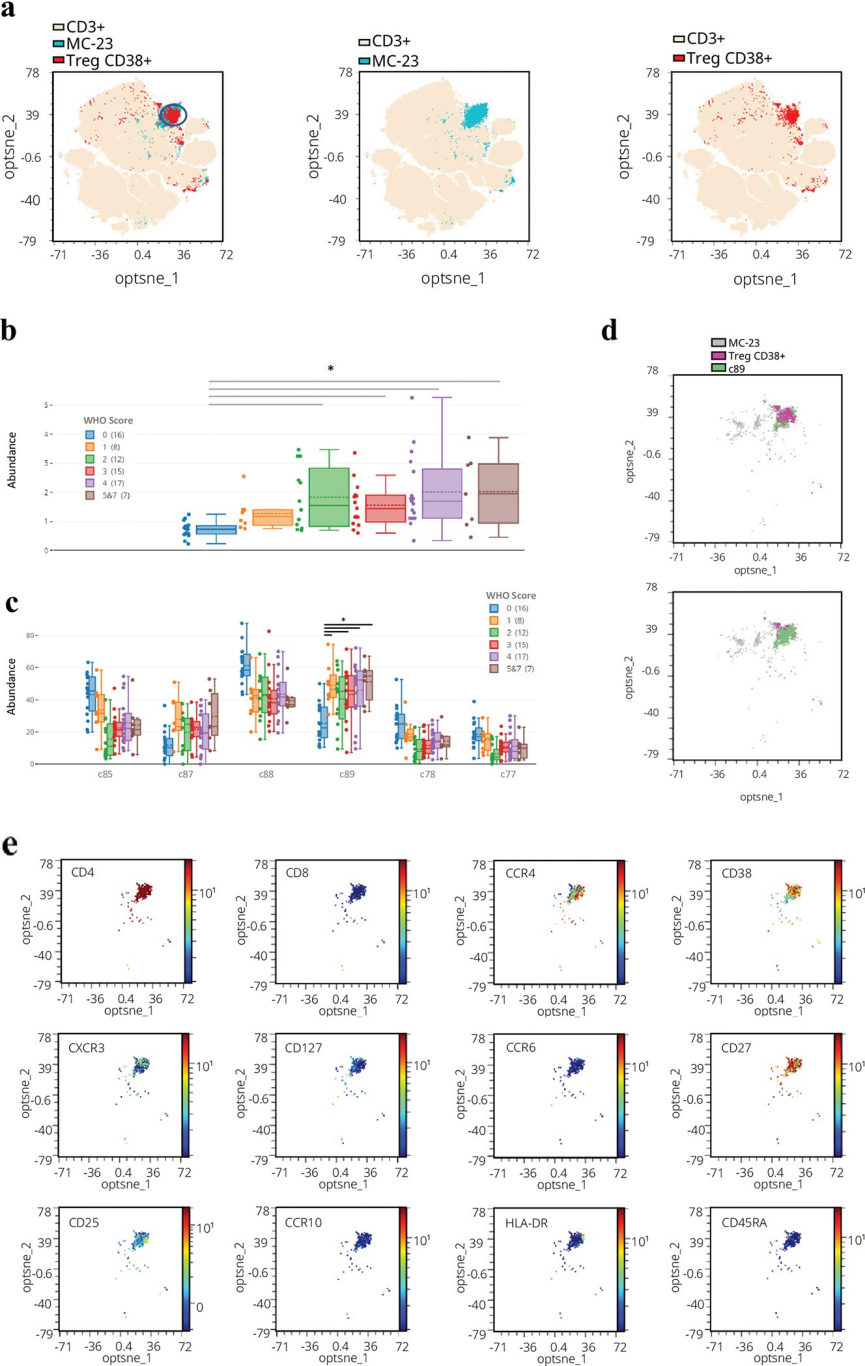
Unsupervised analysis of the T-cell flow cytometry panel. (a) Multidimensional reduced map generated after opt-SNE analysis in CD3+ cells. In light yellow is indicated total CD3+ T cells, in red the CD38+ Treg subset, and in blue the Metacluster 23 (MC-23). The blue circle points to the CD38+ Treg cells. (b) Frequency of MC-23 in each Severity Score group. For each group, the box indicates the lower quartile q1 and the upper quartile q3, and the whiskers the maximum and minimum values. The dotted line indicates the mean, and the continuous line represents each group’s median. (c) Abundances of the 6 clusters by the CITRUS algorithm (gated on total CD3+ T cells) in all the Severity Score groups. Multiple t-tests corrected by multiple comparisons using the Bonferroni-Dunn method. Only statistical significance for the C89 cluster was shown. (d) Clusters are represented in a multidimensional reduced map generated after opt-SNE analysis in CD3+ cells. c89 cluster (green) is found to overlap with CD38+ Treg cells (pink). In the top graph, the CD38+ Treg subset is positioned above the c89. In the bottom graph, c89 is positioned above the CD38+ Treg subset. (e) Intensity expression for every marker in the c89 cluster, with all data from all the individuals, concatenated. High expression is indicated in red, low in blue, and intermediate in cyan-green-yellow.

As observed in a previous section, two cellular subsets can predict a worse outcome for individuals (Fig 1A and 1B). This information may not be easily accessible for clinicians to make decisions about hospitalization as flow cytometry panels are time-consuming and expensive. Nevertheless, from an investigative standpoint, it is intriguing to identify possible cellular subsets that could be associated with disease progression. To perform a deeper analysis of the Treg cells, we used the OMIQ software to perform an unsupervised analysis of the data regarding T-cell subsets. After running the opt-SNE and FlowSOM analysis on total CD3+ T cells, we obtained a multidimensional reduced image where an abundance of metaclusters obtained by the FlowSOM was represented. We identified that the CD38+ Treg cells (determined by manual gating using FMO to determine the positivity and negativity of the signals, S2 Fig) were overlapping in part the metacluster 23 (MC-23), determined by unsupervised analysis (Fig 3A), which was significantly and positively increased in term of abundance (subset percentage regarding total CD3+ T cells) in the most severe scores (Fig 3B). Metacluster 23 was then subsampled and analyzed using the CITRUS algorithm to identify any new subclusters that might be differentially expressed between the groups. From this MC-23, 6 clusters’ abundances were found to be different between Severity Scores (c85, c87, c88, c89, c78 and c77, Fig 3C), especially the c89 cluster showing an abundance increase in all severity Score groups > 1. Moreover, only cluster c89 overlapped almost the entire CD38+ Treg subset (Fig 3D). We studied the expression intensity of each marker in cluster c89, which was found to be CD4+ CD8neg CD127neg and CD25int, indicative of a general Treg phenotype (S3 Fig) and was also described as CD38+ CD27+ CCR6neg CCR10neg HLA-DRneg and CD45RAneg (Fig 3F). Surprisingly, this cluster also presented an intermediate CXCR3 expression, a marker not generally associated with the CD38+ Treg subset (Fig 3F and S3 Fig).

### The frequency of CD38+ Treg but not absolute numbers were positively correlated to the severity score

We were interested in comparing these results with those obtained by manual gating since the CD38+ Treg cell was a cellular subset among the most repeated and important features in all the machine learning models tested, especially in the Elastic Net model, and in the sensitivity analysis.

**Fig 4:**
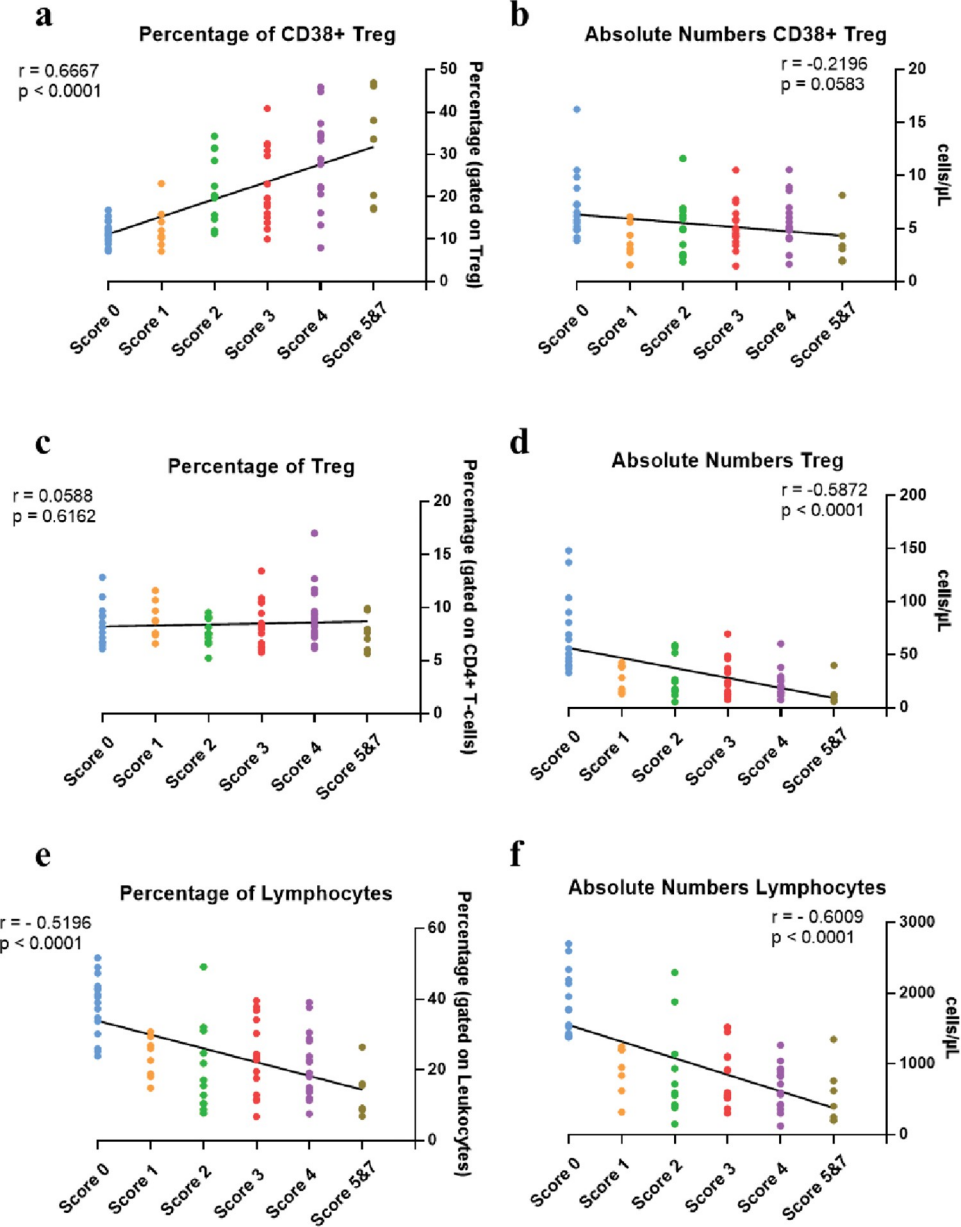
Analysis by manual gating of CD38+ Treg and total Treg cells. Graphs represent individual values and correlation between Severity Score groups and subset percentages (a) and absolute numbers (b) of CD38+ Treg. Percentage (c) and absolute numbers (d) of total Treg cells. Percentage (e) and absolute numbers (f) of total lymphocytes. Spearman correlation was done for each cellular subset. p = p-value and r = correlation coefficient. In bold, when p < 0.05 which is considered as significant.

Attending to the percentage of Treg expressing CD38 in these individuals (defined as CD38+ Treg, gated on total Treg subset), we observed that Score 0 presented a basal frequency of CD38+ Treg, progressively increasing according to the severity score, with a significant and strong positive correlation (r = 0.6667; p < 0.0001, Fig 4A). However, attending to absolute numbers of CD38+ Treg, no significant change was observed regarding the severity (r = -0.2196; p = 0.0583, Fig 4B). We analyzed the percentage and absolute numbers of total Treg cells to determine if the same behavior as CD38+ Treg was observed. On the contrary, the frequency of total Treg (gated on CD4+ T-cells) remained equal in every severity score and non-infected individuals (r = 0.0588; p = 0.6162, Fig 4C). In brief, despite the total Treg absolute numbers decreased with increasing disease severity (r = -0.5872, p < 0.0001, Fig 4D), the CD38+ Treg absolute numbers were maintained (Fig 4B).

The loss of total Treg absolute numbers could be associated with the lymphopenia commonly observed in COVID-19 patients. Indeed, we determined, as already reported, that total lymphocytes in frequency and absolute numbers were diminished in all SARS-CoV-2 infected patients compared with the basal level of the non-infected individuals (Score 0) and were strongly and negatively correlated with the severity score (r = -0.5196; p < 0.0001, Fig 4E, and r = -0.6009; p < 0.0001, Fig 4F). Therefore, the maintained absolute numbers of CD38+ Treg cells and its increase in frequency associated with severity score could be related to a specific role of this specific cellular subset during the disease progression.

Unsupervised analysis, sensitivity analysis and manual gating (flow cytometry) support the machine learning tests discovery and the determination of a cellular subset as a biomarker for the severity of COVID-19.

### An NK cell subset was negatively correlated with the COVID-19 severity

All the machine learning models highlighted that along with CD38+ Treg cells, the other relevant subset related to the severity was the CD16+ CD56neg HLA-DR+ NK subset, where the HLA-DR+ NK subset is related to the adaptive immune response [30]. Among several functions, NK cells are implicated in fighting viral infection and make the connection to the adaptive immune system by producing cytokines and other molecules and are a key cell type in COVID-19 [31].

**Fig 5:**
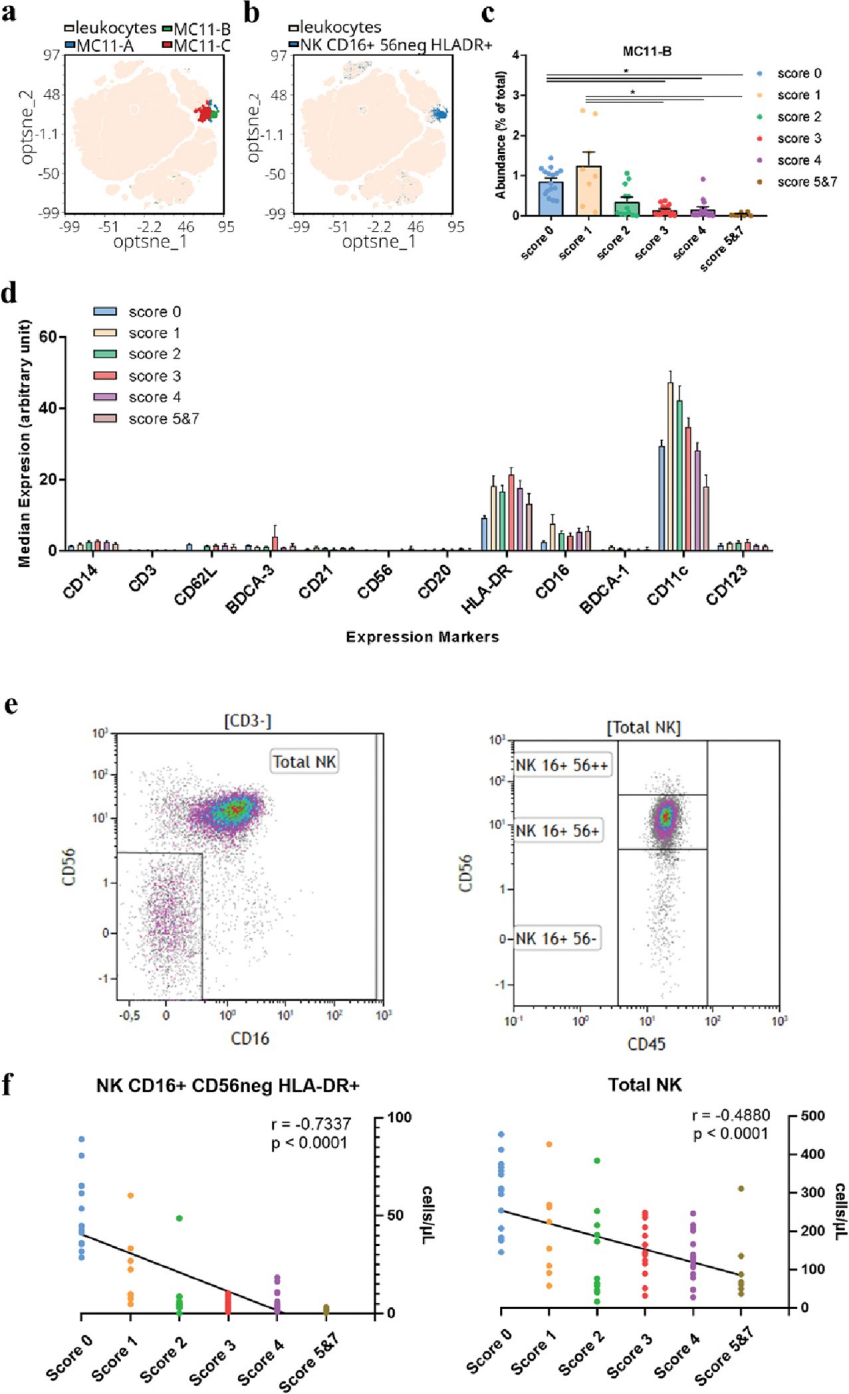
Multi-analysis of the CD16+ CD56neg HLA-DR+ NK subset. (a) Multidimensional reduced map generated after opt-SNE analysis in leukocytes. In light yellow are indicated leukocytes, and MC11-A (blue), MC11-B (green) and MC11-C (red). (b) Multidimensional reduced map generated after opt-SNE analysis in leukocytes. In light yellow are indicated leukocytes and in blue CD16+ CD56neg HLA-DR+ NK cells. (c) Bar graph with individual values, and mean ± SEM of the abundance of MC11-B. 1-way ANOVA, corrected for multiple comparisons using Dunn’s method. *p < 0.05 (d) Mean expression ± SEM of each marker of the NK-DC panel on the CD16+ CD56neg HLA-DR+ NK subset in all Severity Score groups. (e) Dot plots indicate the gating strategy to select total NK cells by positive expression of CD56 or CD16 in CD3neg lymphocytes (left panel). Gated on total NK cells, CD56 expression permits the differentiation of three NK subsets; CD16+ CD56low/neg NK, CD16+ CD56+ NK and CD16+ CD56++ NK cells (right panel). (f) The left panel represents individual values and correlation between Severity Score groups and absolute numbers of CD16+ CD56neg HLA-DR+ NK. The right panel represents individual values and the correlation between Severity Score groups and absolute numbers of total NK Spearman correlation was done for each graph. p = p-value and r = correlation coefficient. In bold, when p < 0.05 which is considered as significant.

We performed the unsupervised analysis on total leukocytes to detect NK cells. After opt-SNE and FlowSOM, the Metacluster 11 (Fig 5A) and especially Metacluster 11B (MC11-B, Fig 5A) was shown to overlap the region where the CD16+ CD56neg HLA-DR+ NK subset was identified (Fig 5B). The MC11-B was significantly different between Score 0 and 1 groups and individuals with score > 1, with a lower CD16+ CD56neg HLA-DR+ NK frequency in higher scores (Fig 5C). As expected, these cells expressed high levels of CD16 and HLA-DR markers but surprisingly, this subset also highly expressed the CD11c marker (Fig 5D).

By manual gating, we defined total NK cells as CD3neg lymphocytes expressing CD56+ or CD16+, excluding the double negative subset CD56neg and CD16neg (Fig 5E, left panel). Then, NK cells can be divided into three subsets; CD16+ CD56low/neg NK, CD16+ CD56+ NK, and CD16+ CD56++ NK, which were already described in the literature (Fig 5E, right panel [32]). Studying the expression of the HLA-DR activation marker on CD16+ CD56neg NK cells, a clear reduction in their absolute numbers was observed (r = -0.7337, p < 0.0001, Fig 5F, left panel). The absolute number of total NK cells showed a similar negative correlation (r = -0.4880, p < 0.0001, Fig 5F, right panel), although the reduction in CD16+ CD56neg HLA-DR+ NK was more drastic. Altogether, the decrease in the absolute numbers of total and specific NK subsets could be related to the general lymphopenia occurring in COVID-19 or even to specific NK cell death induced by the SARS-CoV-2 infection.

Therefore, the unsupervised analysis supported the results obtained by the machine learning model and highlighted the implication of these subtypes as biomarkers to predict COVID-19 severity.

## Discussion

As the pandemic has progressed over the past years, the clinical course of infected patients has been modified, with a reduced hospitalization, severity of disease, and mortality rate. However, the pandemic is not over, and thousands of new infections and deaths are reported daily. Because of this, developing new tools to predict the evolution of COVID-19 patients at the moment of hospitalization and, thus, stratifying individuals into severity score groups could help to focus healthcare efforts on those patients who are expected to have more severe disease. Moreover, facilitating the identification of the specific cellular subsets involved in disease progression could enable the understanding of the pathology’s mechanisms and thus, the development of more effective vaccines and treatments in the future. We propose that the Elastic Net model could be used as an effective machine learning model to predict the Severity Score. Among the several machine learning tested, like Lasso, Random Forest or Kernel Ridge, with the data from our cohort of 75 individuals, we observed that the Elastic Net model had better characteristics than the others, presenting the highest R2 score and the lower MAE and MSE. Moreover, accepting an error of ± 1.5 Severity Score, out of 75 individuals, the Elastic Net correctly predicted the COVID severity in 72 individuals, which means that the model presented high deviations in only 4% of the cases. Unlike the rest of the studies that have developed machine learning models to predict severity as listed in the introduction, in our model, we included healthy individuals as a control of the model and infected asymptomatic or individuals that did not require oxygen therapy. This implementation makes our proposal more robust and helpful than other models because it considers the full spectrum of potential individuals in the pandemic and their specific features. Therefore, this approach could discharge infected patients who do not require oxygen, improving the use of medical equipment and healthcare efforts.

However, we not only wanted to develop a machine learning model capable of predicting the severity score of the different patients but also to help determine immunological features that could be pivotal for the progression of the disease. Among all models tested, several features were highly implicated in predicting severity. Some of them were found previously, for example, the presence of previous comorbidities, especially pulmonary diseases [26, 27], high levels of ferritin [19], or IL-6 levels [17, 24]. Going more in-depth into the immune system, we found 2 specific subpopulations highly correlated with COVID-19 severity.

Regulatory T cells expressing the CD38 marker (CD38+ Treg) was a subset whose percentage increased according to severity, while the absolute number of cells remained stable. Treg is a unique subset of T-cells, responsible for immunological homeostasis, self-tolerance, and prevention and regulation of hyperactivation of the immune system [33–36]. Because of this, it was expected that Treg could be important in COVID-19 severity since mild and severe cases presented hyperinflammation. However, Galvan-Peña *et al*. found that Tregs in severe COVID-19 patients presented a transcriptional signature similar to tumour Tregs, and patients with severe disease presented higher levels of total Treg cells [37]. Moreover, this Treg presented more expression of functional markers like Ki67, CTLA-4, GITR, TCF-1, and higher FOXP3 MFI, in those patients with severe disease compared to mild disease [38]. It has also been described that the IL-10-producing-Treg subset was increased in severe patients and could be responsible for truncating adaptive immune responses, allowing infection to persist and thus causing over-reliance on innate responses [39]. In line with this work, we showed that IL-10 concentration in plasma was positively correlated with the more severe score individuals. Moreover, our results align with those observed by Søndergaard *et al*. [40], where Treg expressing CD38 marker were increased related to COVID-19 severity. The Treg-expressing CD38 subset has been described as a Treg subset with a high immunosuppressive ability [41, 42], and the expression of CD38, in this case, could explain the activation of the Treg and complete the list of functional markers described by Vick *et al*. [38]. Also, Vick *et al*. described that these Treg had also increased expression of CXCR3, a marker needed to migrate to tissues [38]. We also detected this marker in the CD38+ Treg subset through the unsupervised algorithm. Altogether, this could indicate that severe patients have activated Tregs in the blood, with levels increased according to severity. However, it was interesting to note that CXCL10 and CXCL9 were found to increase in peripheral blood in severe patients and could retain the retention of the CXCR3+ Treg in the periphery, preventing the migration to tissues [38].

Regarding NK cells, CD16+ CD56neg HLA-DR+ NK absolute numbers decreased when the COVID-19 severity increased. CD16+ CD56neg NK is a type of NK cell with a mature and cytotoxic phenotype implicated in resolving viral infections [31, 43]. However, some studies demonstrated that these NK cells overexpressed several markers related to a dysfunctional phenotype in severe COVID-19, like CD39 [44], PD-1 [44, 45], NKG2A [44, 46], DUSP2 [47], CD69 [45, 47], CD38 [47], LAG-3 [45] and TIM-3 [45]. Moreover, these cells are also described as hyporesponsive to the production of TNF-α, IFN-γ, IL-2 and granzyme, related to their cytotoxic function [48, 49]. We did not analyse these markers in our cohorts, but it would be interesting to analyse the presence of these markers as related to a dysfunctional phenotype. Additionally, HLA-DR was described as a functional marker in NK cells by Erokhina *et al*. [30, 50], and HLA-DR+ NK cells were presented to produce proinflammatory cytokines, degranulate, and easily proliferate in response to stimuli. The loss of such a subset could be due to a massive cell death after performing their function. One can assume that in severe COVID-19 individuals with hyper-inflammation, those who have exhibited a higher level of functionality and therefore, higher levels of pro-inflammatory response against the virus, may show lower levels of presence in the periphery.

Aranami *et al*. [51] described that patients with Multiple Sclerosis with NK expressing high levels of CD11c had a better remission than patients with lower levels of CD11c, suggesting that CD11c is a functional NK marker too. In this work, we observed an increase in scores 1, 2 and 3 but a decrease in score 5&7 individuals, showing that a higher level of CD11c on CD16+ CD56neg HLA-DR+ NK could predict a less severe progression and the loss of this marker could predict a more severe progression. Therefore, the decrease in the expression of this marker regarding severity could indicate that NK cells are dysfunctional or experience selective cell death. Altogether, this work supports the idea that reduced levels of CD16+ CD56- HLA-DR+ NK cells in those that will experience a more severe COVID-19 could be due to the fact that the SARS-CoV-2 infection triggers alterations in NK and, subsequently, the dysfunction and apoptosis of these cells.

One of the major limitations of the study is the heterogeneity of the individuals included in this study. It could be worth considering that individuals who present at the hospital early in the course of their infection could receive prompt medical attention and thus be less susceptible to developing a severe form of the disease. Nevertheless, the patients further included in WHO score 2 presented themself to the hospital with a median of 8 days after symptoms onset and those in WHO score 5–7 waited around 5 days, even if not significantly different between severity groups (Table 2). This data implies that the patient terminating in the less severe score did not get to the hospital in the early phase of the infection. Even though this fact could be an important confounding factor, this work aimed to identify those who were at risk of developing the severe disease at the time of Urgency Service screening to assist physicians in determining which patients should be hospitalized based on the relevant biomarkers regardless of the symptoms they may present. Another limitation of this work is the low number of individuals included since the number of patients in some scores is reduced, preventing the machine learning models from correctly learning these minority cohorts. Therefore, including more individuals from all Severity Score groups, especially in Scores 1 and 5&7, could improve the model and make it more precise.

It is important to note that using a comprehensive flow cytometry analysis is a very tedious, expensive and time-consuming technique, and it is not employed in the clinical routine. Although this data cannot easily be used in other models, we observed that in our machine model, the best performance was reached when all the variables of the study were included (clinical, personal, and flow cytometry data), highlighting the importance of the flow cytometry data. In addition, the use of cytometry data helped us to identify immune subpopulations related to the severity of COVID-19 that could be valuable for investigating the underlying mechanisms by which the presence or absence of these subsets may impact the clinical evolution of patients.

With this work, we present a machine learning model that could help to stratify mild or severe COVID-19 patients, as previously published, at the hospitalization time. Even though this model may not be applicable in a clinical setting, it can aid researchers in evaluating which immunological subsets may contribute to protection against severe progression, as well as those that may be associated with disease evolution. Moreover, we proposed two cellular subsets as the better features to stratify patients according to severity, beyond biochemical parameters, that could help to better understand the induction and progression of the severity symptoms in COVID-19.

## Materials and methods

### Ethics statement

Written informed consent was obtained under the Declaration of Helsinki Protocol. According to their guidelines, the "Comité de Ética de la Investigación con Medicamentos (CEIm) del Hospital General Gregorio Marañón" approved the study (COV1-20-007).

### Patients and blood samples

Blood samples were obtained from 75 individuals, 16 of them were healthy donors, and 59 were infected by SARS-CoV-2 between September 2020 and August 2021 when they were admitted to the Urgency Service at the General University Hospital Gregorio Marañón, Madrid, Spain. SARS-CoV-2 infection was confirmed with a positive test (PCR or antigen test). The clinical data and peripheral blood samples in our study were collected at the time of presentation at the Emergency Service during the COVID-19 and non-COVID-19 patient screenings done by PCR or antigen test. Individuals were invited to participate in the study and provided their consent before blood samples were taken. Subsequently, patients were hospitalized or discharged to recover at home based on their symptoms. The WHO scores were evaluated retrospectively based on the clinical evolution of hospitalized patients. The blood samples were processed within the first 18 hours. The clinical course of each patient was obtained through the Health Care Information System (HCIS). The maximum oxygen therapy required during the hospitalisation was used to classify patients into eight different cohorts, detailed in Table 4, adapted from the classification of the World Health Organization (WHO, [52]). The symptoms listed in Table 2 correspond to the symptoms developed since the onset of the symptoms to the day of hospital admission, via a questionnaire, and not just the symptoms that they presented on the day of Hospital arrival. The listed symptoms are not a recollection of the symptoms that they experienced during their hospitalization. Detailed information about patients’ characteristics is provided in Table 2 and the data collection, including clinical characteristics and flow cytometry analysis, can be found in S2 Table. Since none of the patients needed intubation or mechanical ventilation, Score 6 was not represented. Because of the reduced number of individuals in Scores 5 and 7, we decided to group all the patients in Score 5&7 group.

**Table 4:** Classification of individuals according to oxygen therapy during COVID-19. Individuals were classified with an adaptation of the WHO Severity Score into 8 cohorts. Score 0 refers to the non-infected individuals. The remaining scores refer to individuals infected by SARS-CoV-2. From Score 1 to 7, the highest oxygen requirement is described according to the highest Severity Score.

Score 0	No SARS-CoV-2 infection
Score 1	SARS-CoV-2 infection without limitation of ordinary activities
Score 2	SARS-CoV-2 infection hospitalised but without oxygen therapy
Score 3	SARS-CoV-2 infection hospitalised with maximum oxygen therapy of 2L
Score 4	SARS-CoV-2 infection hospitalised with minimum oxygen therapy of 3L
Score 5	SARS-CoV-2 infection hospitalised with non-invasive ventilation or high flow ventilation
Score 6	SARS-CoV-2 infection hospitalised with intubation or mechanical ventilation
Score 7	SARS-CoV-2 infection hospitalised leading to death

### Cell surface marker staining and cytokines analysis in plasma

Whole blood samples were labelled for surface markers with the fluorochrome-labelled antibodies distributed in four cytometry panels to detect 150 subsets of innate and adaptive cells, besides their differentiation and activation status, following the already published protocols [53]. Whole blood was stained with an antibody mix for each panel, and after incubation, red blood cells were lysed using RBC Lysis/Fixation Solution (Bio-Legend). After the lysis, stained blood was analysed by flow cytometry using a MACSQuant Analyser 16 cytometer (Miltenyi Biotec). Flow cytometry gating strategy for each panel is detailed in S4–S7 Figs (S4 Fig: a gating strategy for T lymphocytes panel; S5 Fig: a gating strategy for B lymphocytes panel; S6 Fig: a gating strategy for innate cells panel; and S7 Fig: a gating strategy for T lymphocytes panel 2). For controversial marker signals, Fluorescence Minus One (FMO) were done and listed in S2A Fig for T- and Tfh-Tgd-lymphocytes panels, S2B Fig for B-lymphocytes panel, and S2C Fig for innate immune cells’ panel.

With the rest of the whole blood, we centrifuge it and keep plasma at -80°C until their analysis. Cytokines in plasma were analysed using the microfluidic ELISA equipment ELLA-Protein Simple (Biotechne), measuring the concentration of cytokines in two different cartridges, the first cartridge could evaluate 4 cytokines simultaneously (IL-1b, IL-6, IL-8, and TNF-α) and the second cartridge could evaluate 8 cytokines simultaneously (CCL2, IL-12p70, IL-10, IL-13, IL-17A, CXCL10, GM-CSF and IFN-γ).

### Data pre-processing

For each of the 75 individuals, a total of 575 variables were collected containing patient characteristics and their blood sample information (S1 Table). In order to adequately process the available patient data with any machine learning model, we have had to apply several pre-processing steps. In particular, we first one-hot encoded the categorical variables (Blood Type and Ethnicity). Later we imputed missing data, a prevalent problem among collected features: 2 variables presented over 60% missing data, 9 variables between 30–50%, 22 variables between 20–30%, and 4 variables between 1–2%. For this purpose, for binary variables, the most frequent value expressed in the variable range was used as the imputation estimate and for numeric variables, we used K-NN imputation if the numeric variable to be imputed was strongly correlated (over 0.7 absolute correlation rate) with other variables and, otherwise, the imputation was carried out with the mean of the available data for the variable to be imputed. Finally, to homogenise the range of all variables, non-binary variables were normalised to zero mean and unitary standard deviation. The sensitivity analysis of the features using the pytolemaic toolbox (https://pypi.org/project/pytolemaic/).

### Machine learning model

To automatically predict COVID-19 severity, we have used an Elastic Net regression model [54]. Although the problem to be solved is to classify patients into six cohorts, we have posed the problem as a regression problem with values between 0 and 7.

Additionally, we have thought it appropriate to use a linear model, since we have a problem with 575 variables or input features and no more than 75 patients, so it is possible to find a linear solution to it, and using nonlinear models can lead to overfitting. Furthermore, the fact of using an Elastic Net that incorporates an L1 regularisation gives us an additional advantage, as it eliminates unnecessary variables and allows us to automatically detect the factors that differentiate patients between different cohorts.

To evaluate these advantages, in the experimental analysis, we have included other baseline methods, such as Kernel Ridge regression (with both linear and RBF kernel), Lasso regression, Random Forest, and linear Gaussian Process, which can provide linear and nonlinear solutions and feature relevance/selection capabilities. All the models under study have been evaluated with a Leave One Out (LOO) train/test scheme and an inner LOO has been included for the cross-validation of the model parameters. For the Elastic Net, Kernel Ridge and Lasso Regression models, alpha values in the set [0.01, 0.1, 1, 100, 1000] were cross-validated. In addition, for the Elastic Net model, L1 ratio values in the set [0.1, 0.3, 0.5, 0.7, 0.9] were cross-validated. In the case of the Random Forest model, maximum tree depth values in the set [2, 4, 8, 16, 32] were cross-validated.

### Unsupervised flow cytometry analysis setting

Cytometry data was used to perform a deeper analysis using OMIQ software (https://www.omiq.ai/). For the analysis of the T-cell panel, unsupervised analyses were done on CD3+ T-cells. After the compensation and scaling of all markers in this panel, 330.000 CD3+ lymphocyte events were selected proportionally for each Score group (Score 0, 1, 2, 3, 4 and 5&7). Later, we performed the algorithm opt-SNE, a modified version of the t-SNE (t-distributed Stochastic Neighbour Embedding) that allows characterising high-dimensional data into two dimensions. Opt-SNE settings were: Max Iterations; 1000, opt-SNE End; 5000, Perplexity; 30, Theta; 0.5, Components; 2, Verbosity; 25, with Random Seed. After opt-SNE, we performed a FlowSOM, a clustering algorithm that generates metaclusters; grouping cells sharing similitudes. FlowSOM settings were: xdim; 15, ydim: 15, rlen; 100, running elbow metaclustering. CITRUS (cluster identification, characterization, and regression) is an algorithm for the fully automated discovery of statistically significant clusters, stratifying biological signatures. CITRUS settings were: Min Cluster Size Percent; 0.05, Cross Validation Folds; 1, Regression Methods; pamr, sam.

We also compensated and scaled the markers in the panel of innate immune cells, and the unsupervised analyses were done on gated leukocytes. 450.000 leukocyte events were analysed under proportional sampling between groups (Score 0, 1, 2, 3, 4 or 5&7). Later, we performed opt-SNE with the following settings: Max Iterations; 7000, opt-SNE End; 7000, Perplexity; 100, Theta; 0.5, Components; 2, Verbosity; 25, with Random Seed. After opt-SNE, we performed FlowSOM with settings: xdim; 10, ydim: 10, rlen; 100, running elbow metaclustering. CITRUS settings were: Min Cluster Size Percent; 0.01, Cross Validation Folds; 1, Regression Methods; pamr, sam.

The abundance, in the unsupervised methods, refers to the frequency of clusters, metaclusters or cellular subsets, regarding the total events analysed.

### Software and statistical analysis

Flow cytometry data were analysed using Kaluza software (version 2.1, Beckman Coulter). Graphs were made using GraphPad Prism (version 8.0, GraphPad). Different machine learning models, as well as data pre-processing, were implemented using Python 3.7 and, in particular, using sklearn library [55]. Statistical analyses of cellular subsets were conducted using GraphPad Prism and SPSS (version 25.0, IBM). Graphs represented mean value ± SEM (Standard Error of the Mean). Each figure legend describes the specific statistical test used to evaluate the experiments.

## Supporting information

S1 Fig 30 most important features were identified through sensitivity analysis out of a total of 575 features. The mean sensitivity of the 30 most relevant features was shown among the entire set of 575 features. The two cellular subsets that were identified as determinants across all the machine learning models tested are highlighted with orange rectangles. Out of the 575 features tested, 419 were considered non-relevant, and 156 were deemed relevant, with the 30 most relevant features displayed on the graph.
(PPTX)

S2 Fig Fluorescence Minus One (FMO) controls for markers in the four flow cytometry panels. Classical staining is represented in black, and FMO staining for each marker is indicated in red. For T lymphocytes and Tfh-Tgd panels (A), gates were set in total CD3+ lymphocytes. For the B lymphocyte panel (B), gates were set in total lymphocytes. For the innate immune cells’ panel (C), gates were set on total leukocytes, except for BDCA-3 FMO, which was analysed on total DCs.
(PPTX)

S3 Fig The intensity of expression of cellular markers. Intensity expression for every marker in the CD38+ Treg subset, with all data from all the individuals, concatenated. High expression is indicated in red, low in blue, and intermediate in cyan-green-yellow.
(PPTX)

S4 Fig Traditional manual gating strategy for the analysis of the T-cell panel. A representative example of flow cytometry dot plots determined from whole blood labelled from one individual in the study is represented. The subsets, including Naive/CMem/EMem/TemRA subsets, as well as CD38 and HLA-DR expression, were analyzed in CD4+, CD8+ T lymphocytes and Treg cells.
(PPTX)

S5 Fig Traditional manual gating strategy for the analysis of the B-cell panel. A representative example of flow cytometry dot plots and histograms determined on whole blood labelled from one individual in the study is represented. The entire B-cell analysis was conducted in the CD3neg lymphocytes CD19+ CD20+ gate. For the activation status, CD25+, CD80+/CD86+, and CD25+ CD80+/CD86+ expressions were analyzed for each population. Within CD25+, CD80+/CD86+, and CD25+ CD80+/CD86+ subpopulations, PD-1 expression was also analyzed.
(PPTX)

S6 Fig Traditional manual gating strategy for the analysis of the innate immune cells’ panel. A representative example of flow cytometry dot plots and histogram determined from whole blood labelled from one individual in the study is represented. Dendritic cells were analyzed as a LIN- HLA-DR+ subset, where LIN- refers to total leukocytes that are negative for CD3+, CD14+, CD20+ and CD56+ markers.
(PPTX)

S7 Fig Traditional manual gating strategy for the analysis of the Tfh-Tgd panel. A representative example of flow cytometry dot plots and histograms determined from whole blood labelled from one individual in the study is represented. Tfh cells were analyzed within the CD4+ T lymphocytes. CD28, TCRgd and PD-1/ICOS expression were analyzed in CD4+, CD8+ and CD4/CD8 double positive and double negative T-lymphocyte subsets.
(PPTX)

S1 Table Variables collected for the study. (XLSX)

S2 Table Clinical and Flow Cytometry data for all individuals. (XLSX)
