# Supplementary figures and images for: Multidimensional analysis of immune cells from COVID-19 patients identified cell subsets associated with the severity at hospital admission
Source: PLoS Pathog. 2023 Jun 13;19(6):e1011432. doi: 10.1371/journal.ppat.1011432 (PMC10263360; doi:10.1371/journal.ppat.1011432)

## Slide 1
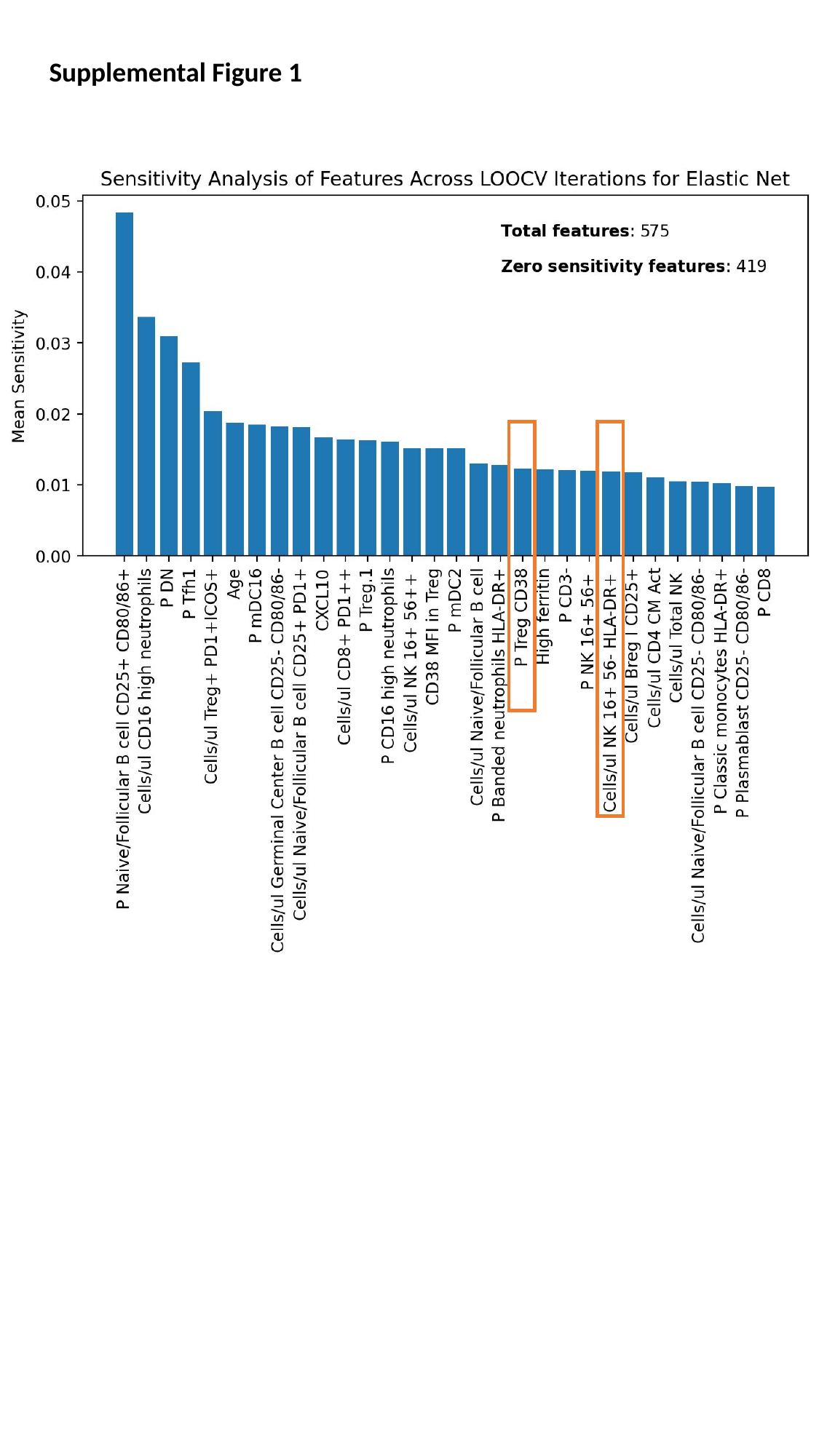

Supplemental Figure 1

Supplement: S1 Fig — The mean sensitivity of the 30 most relevant features was shown among the entire set of 575 features. The two cellular subsets that were identified as determinants across all the machine learning models tested are highlighted with orange rectangles. Out of the 575 features tested, 419 were considered non-relevant, and 156 were deemed relevant, with the 30 most relevant features displayed on the graph. (PPTX) [file ppat.1011432.s001.pptx]

## Slide 1
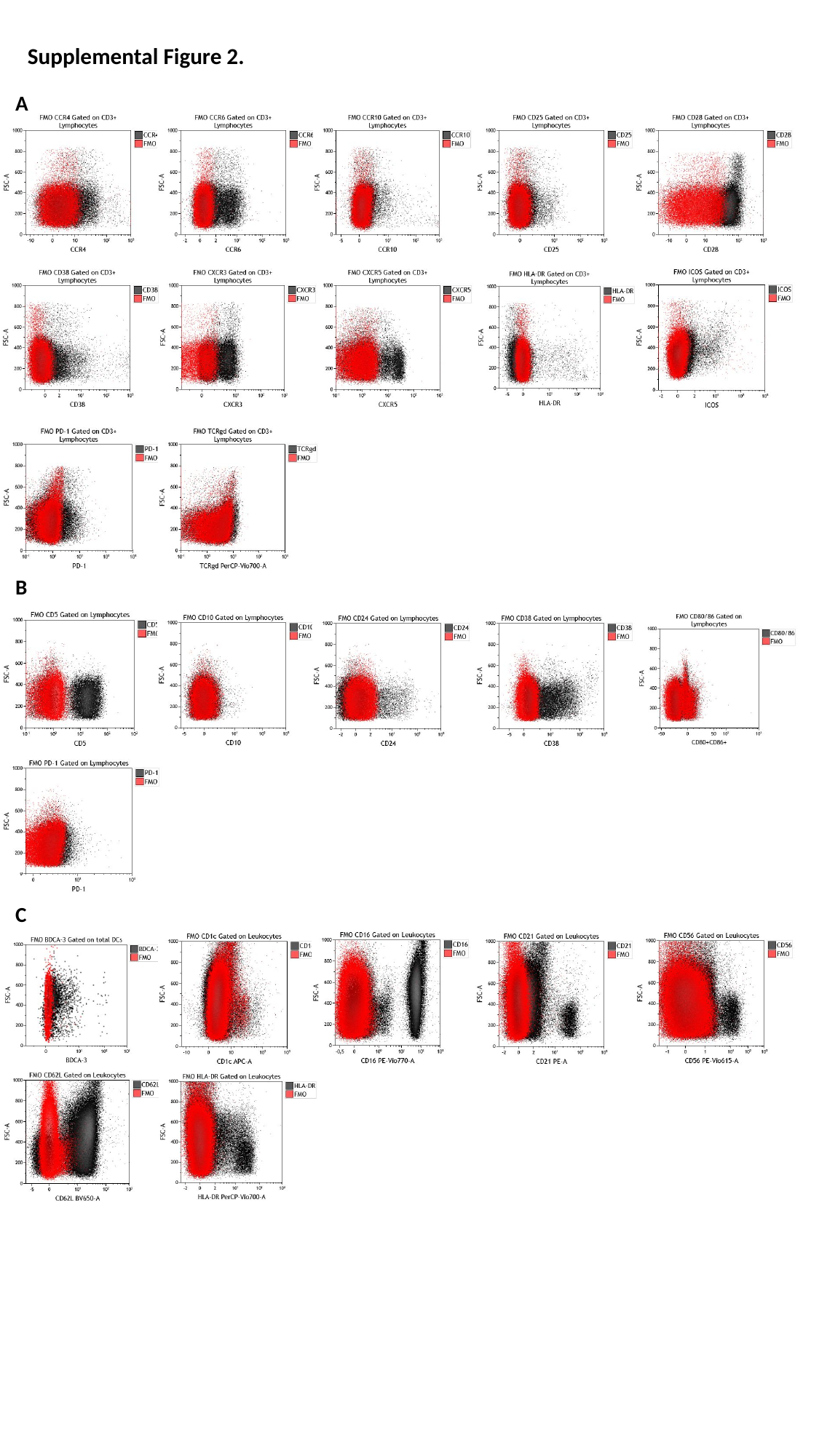

Supplemental Figure 2.
A
B
C

Supplement: S2 Fig — Classical staining is represented in black, and FMO staining for each marker is indicated in red. For T lymphocytes and Tfh-Tgd panels (A), gates were set in total CD3+ lymphocytes. For the B lymphocyte panel (B), gates were set in total lymphocytes. For the innate immune cells’ panel (C), gates were set on total leukocytes, except for BDCA-3 FMO, which was analysed on total DCs. (PPTX) [file ppat.1011432.s002.pptx]

## Slide 1
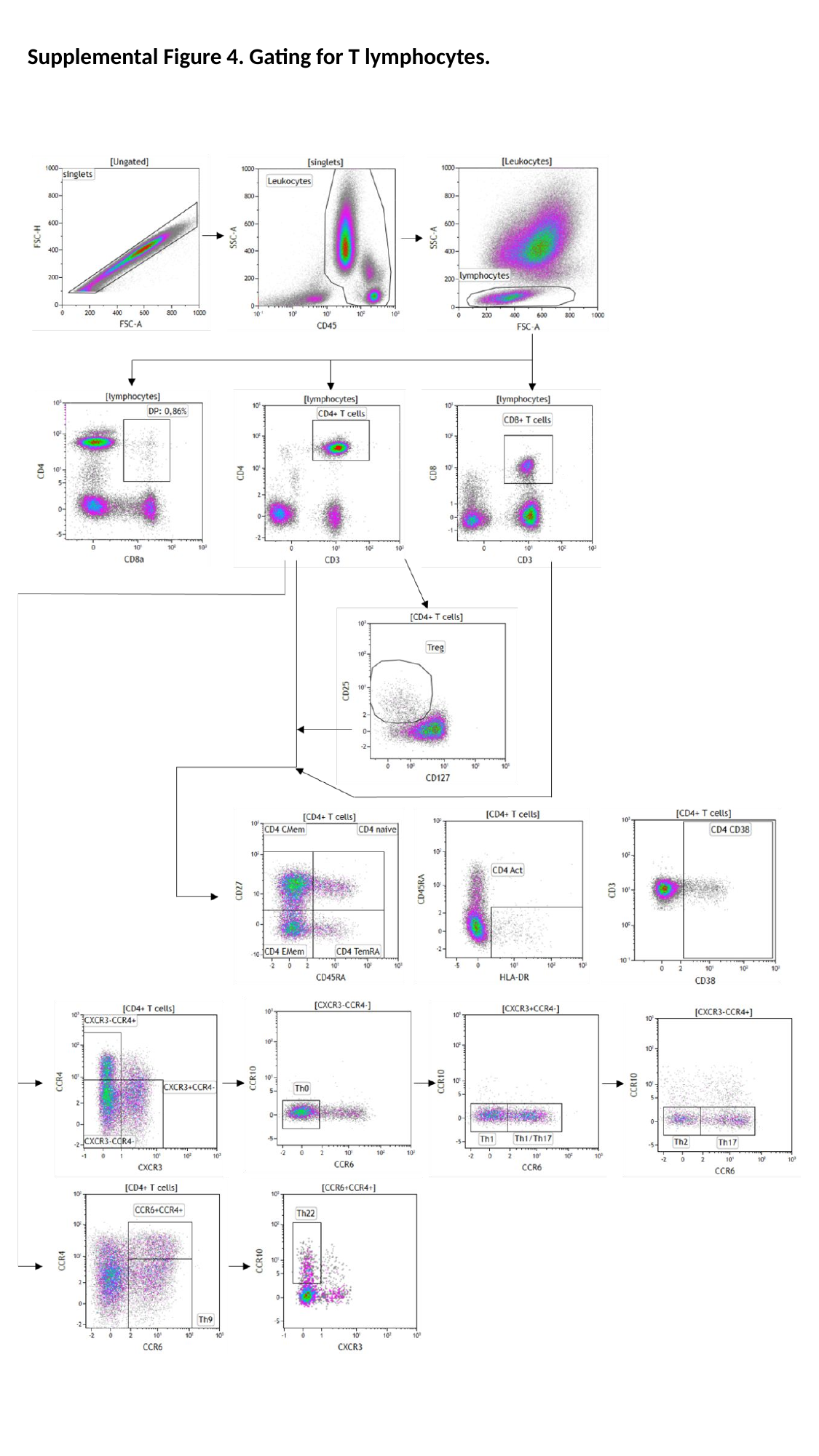

Supplemental Figure 4. Gating for T lymphocytes.

Supplement: S4 Fig — A representative example of flow cytometry dot plots determined from whole blood labelled from one individual in the study is represented. The subsets, including Naive/CMem/EMem/TemRA subsets, as well as CD38 and HLA-DR expression, were analyzed in CD4+, CD8+ T lymphocytes and Treg cells. (PPTX) [file ppat.1011432.s004.pptx]

## Slide 1
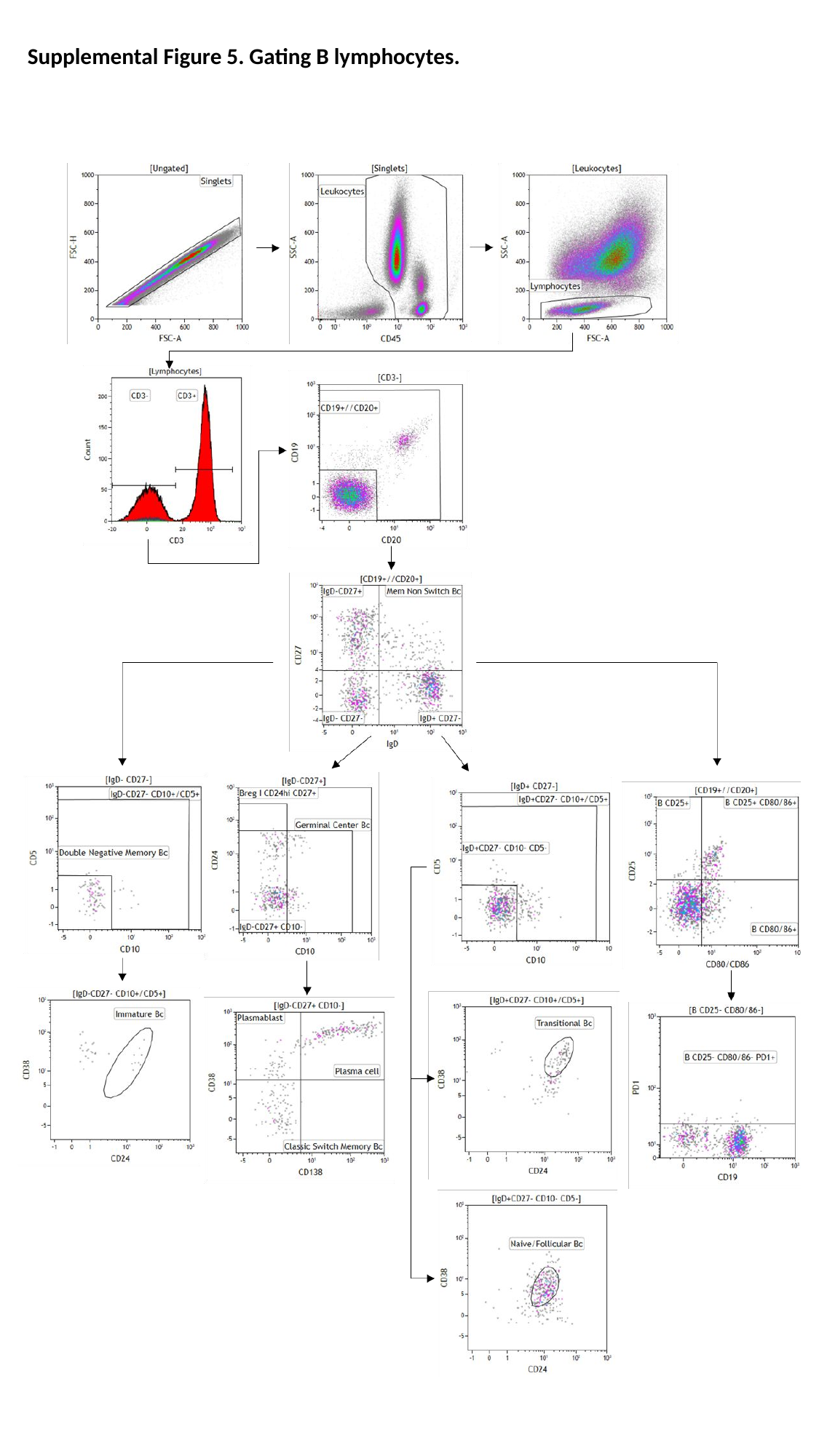

Supplemental Figure 5. Gating B lymphocytes.

Supplement: S5 Fig — A representative example of flow cytometry dot plots and histograms determined on whole blood labelled from one individual in the study is represented. The entire B-cell analysis was conducted in the CD3neg lymphocytes CD19+ CD20+ gate. For the activation status, CD25+, CD80+/CD86+, and CD25+ CD80+/CD86+ expressions were analyzed for each population. Within CD25+, CD80+/CD86+, and CD25+ CD80+/CD86+ subpopulations, PD-1 expression was also analyzed. (PPTX) [file ppat.1011432.s005.pptx]

## Slide 1
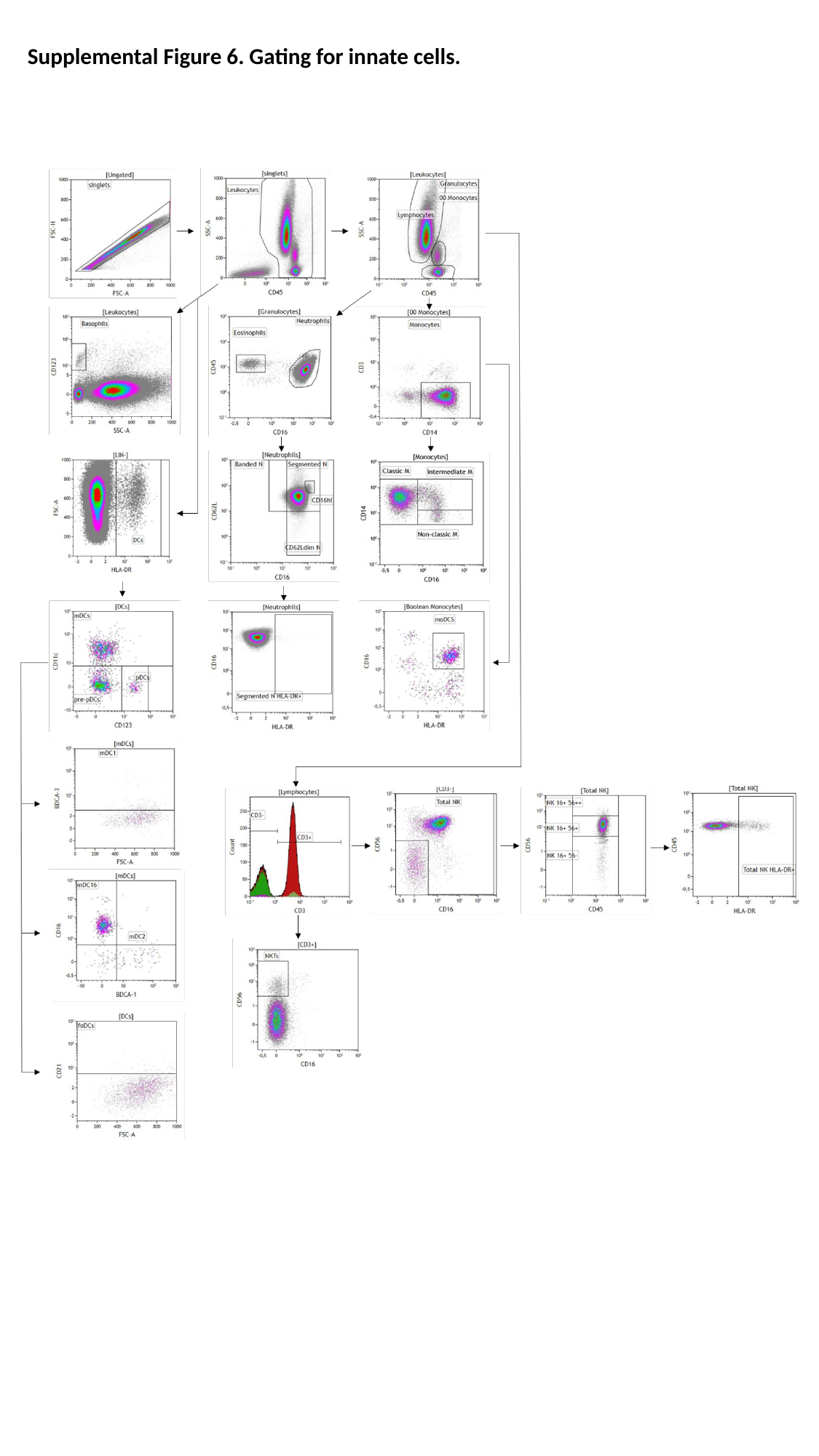

Supplemental Figure 6. Gating for innate cells.

Supplement: S6 Fig — A representative example of flow cytometry dot plots and histogram determined from whole blood labelled from one individual in the study is represented. Dendritic cells were analyzed as a LIN- HLA-DR+ subset, where LIN- refers to total leukocytes that are negative for CD3+, CD14+, CD20+ and CD56+ markers. (PPTX) [file ppat.1011432.s006.pptx]

## Slide 1
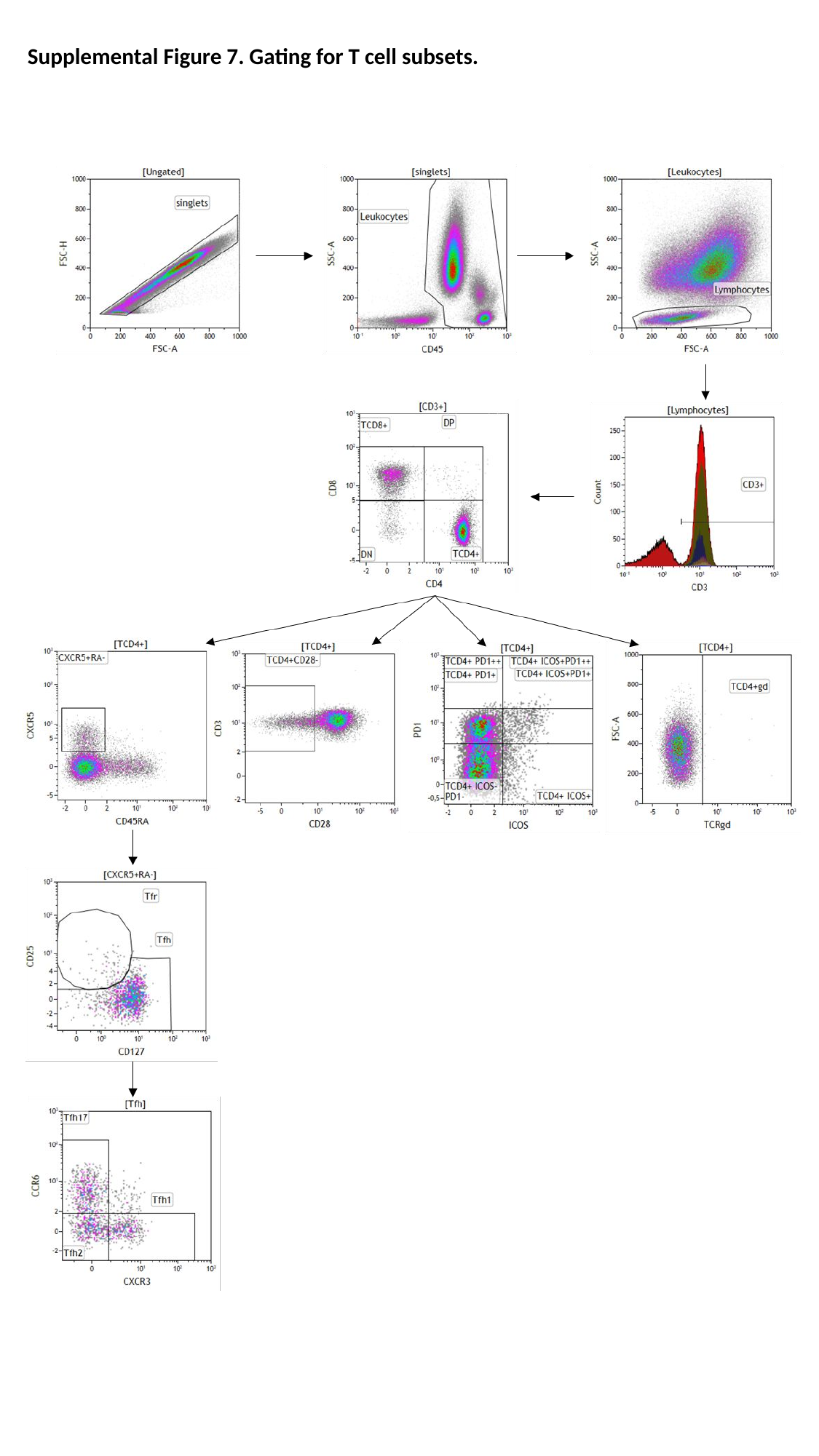

Supplemental Figure 7. Gating for T cell subsets.

Supplement: S7 Fig — A representative example of flow cytometry dot plots and histograms determined from whole blood labelled from one individual in the study is represented. Tfh cells were analyzed within the CD4+ T lymphocytes. CD28, TCRgd and PD-1/ICOS expression were analyzed in CD4+, CD8+ and CD4/CD8 double positive and double negative T-lymphocyte subsets. (PPTX) [file ppat.1011432.s007.pptx]
