# Supplementary material for: Multidimensional analysis of immune cells from COVID-19 patients identified cell subsets associated with the severity at hospital admission
Source: PLoS Pathog. 2023 Jun 13;19(6):e1011432. doi: 10.1371/journal.ppat.1011432 (PMC10263360; doi:10.1371/journal.ppat.1011432)
Supplement: S3 Fig — Intensity expression for every marker in the CD38+ Treg subset, with all data from all the individuals, concatenated. High expression is indicated in red, low in blue, and intermediate in cyan-green-yellow. (PPTX) [file ppat.1011432.s003.pptx]

## Slide 1
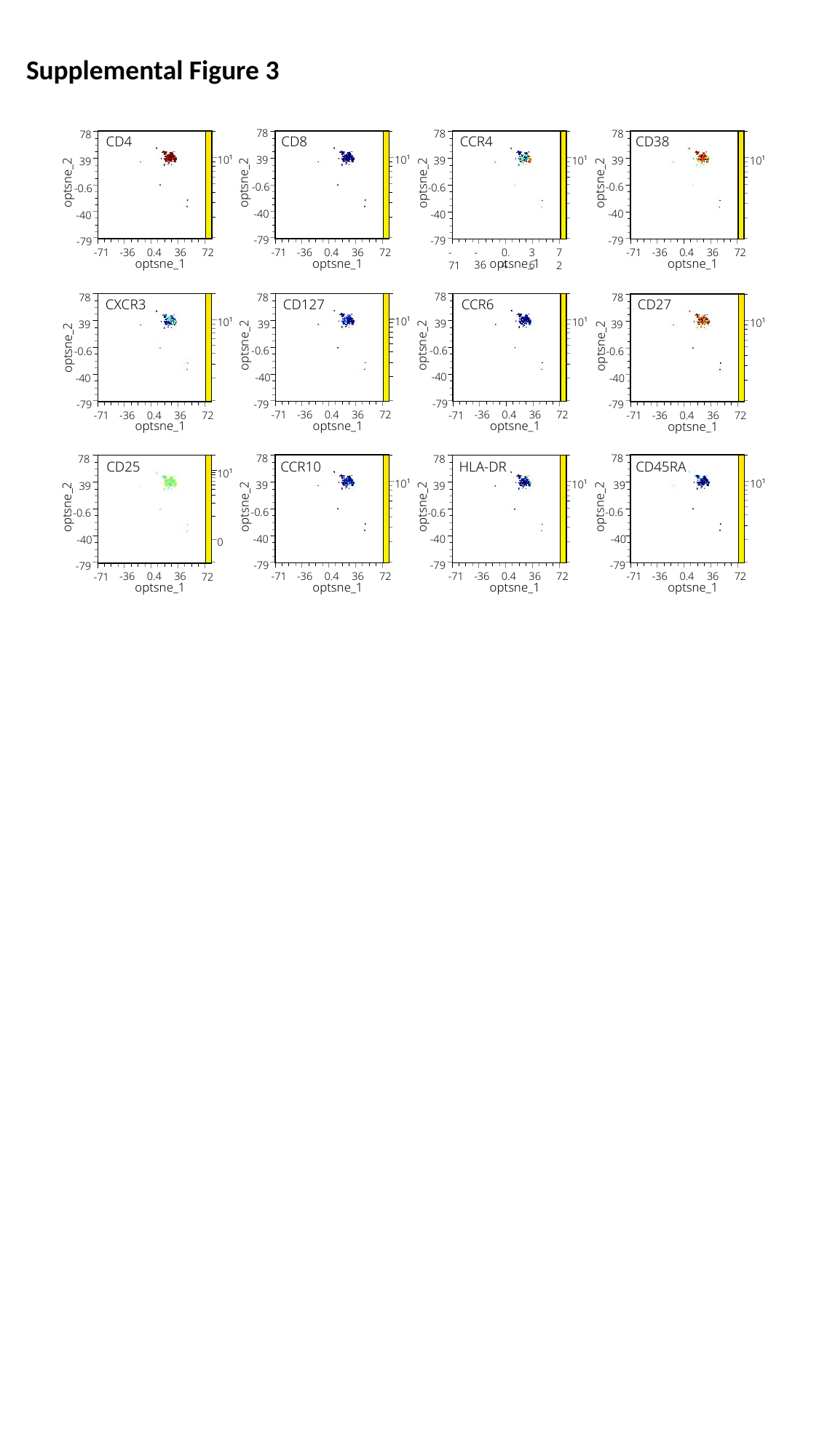

Supplemental Figure 3
78
39
-0.6
-40
-79
10¹
optsne_2
-36
36
0.4
72
-71
optsne_1
78
39
-0.6
-40
-79
10¹
optsne_2
-36
36
0.4
72
-71
optsne_1
78
39
-0.6
-40
-79
10¹
optsne_2
-36
36
0.4
72
-71
optsne_1
78
39
-0.6
-40
-79
10¹
optsne_2
-36
36
0.4
72
-71
optsne_1
CD38
CD8
CD4
CCR4
78
39
-0.6
-40
-79
10¹
optsne_2
-36
36
0.4
72
-71
optsne_1
78
39
-0.6
-40
-79
10¹
optsne_2
-36
36
0.4
72
-71
optsne_1
78
39
-0.6
-40
-79
10¹
optsne_2
-36
36
0.4
72
-71
optsne_1
78
39
-0.6
-40
-79
10¹
optsne_2
-36
36
0.4
72
-71
optsne_1
CCR6
CD27
CD127
CXCR3
78
39
-0.6
-40
-79
10¹
optsne_2
-36
36
0.4
72
-71
optsne_1
78
39
-0.6
-40
-79
10¹
optsne_2
-36
36
0.4
72
-71
optsne_1
78
39
-0.6
-40
-79
10¹
optsne_2
-36
36
0.4
72
-71
optsne_1
78
39
-0.6
-40
-79
10¹
0
optsne_2
-36
36
0.4
72
-71
optsne_1
CCR10
CD25
CD45RA
HLA-DR
